# Chromosome instability and benefit from adjuvant anthracyclines in breast cancer

**DOI:** 10.1038/bjc.2012.232

**Published:** 2012-05-29

**Authors:** A F Munro, C Twelves, J S Thomas, D A Cameron, J MS Bartlett

**Affiliations:** 1 Endocrine Cancer Group, Edinburgh Cancer Research UK Centre, MRC IGMM, University of Edinburgh, Crewe Road South, Edinburgh EH4 2XR, UK; 2 University of Leeds and Cancer Research UK Centre, St James’ University Hospital, Leeds LS2 9JT, UK; 3 Edinburgh Cancer Centre, Western General Hospital, Crewe Road South, Edinburgh EH4 2XU, UK; 4 Transformative Pathology, Ontario Institute for Cancer Research, MaRS Centre, South Tower, 101 College Street, Suite 800, Toronto, Ontario, Canada M5G 0A3

**Keywords:** breast cancer, epirubicin, biomarkers, chromosome instability, fluorescent *in situ* hybridisation (FISH)

## Abstract

**Background:**

Duplication of the centromeric region of chromosome 17 (Ch17CEP) is associated with sensitivity to anthracyclines. An explanation may be chromosome instability (CIN); a frequent event in solid tumours associated with poor outcome. The predictive value of CIN seems to be drug dependent and CIN has been associated with both sensitivity and resistance to chemotherapy.

**Methods:**

In this study, we used fluorescent *in situ* hybridisation for chromosomes 1, 7, 11, 17 and 18 to identify patients with high tumour CIN% in 322 patients recruited into the BR9601 clinical trial.

**Results:**

High tumour CIN% was correlated to Ch17CEP (*P*=3.68e−7) and is associated with a reduced RFS (*P*=0.0011) and OS (*P*=0.04). Patients with high CIN had a decreased risk of death on E-CMF compared with CMF.

**Conclusion:**

CIN is of prognostic significance and may be of predictive value in determining anthracycline response, although further testing is required.

Chromosome instability (CIN) is a frequent characteristic of solid tumours and is characterised by gain or loss of whole or partial chromosomes. Patients whose tumours have CIN have a poor prognosis across many cancer types including breast and colon cancer (Carter *et al*, 2006; Walther *et al*, 2008; Smid *et al*, 2010). Chromosome instability is more prevalent in ER-negative disease, basal and triple-negative breast tumours (Smid *et al*, 2010).

We recently demonstrated that duplication of the centromeric region of chromosome 17 (Ch17CEP duplication), one of several chromosomes utilised to assess CIN (Takami *et al*, 2001; Miyoshi *et al*, 2002), is a predictive marker of sensitivity to anthracycline-containing chemotherapy in a meta-analysis of NEAT, BR9601 and MA.5 clinical trials (Bartlett *et al*, 2009, 2010). Initial analysis of the NEAT, BR9601 and MA.5 studies assessed the predictive role of HER2 amplification on outcome in patients treated with anthracycline-containing polychemotherapy compared with CMF gave conflicting results (Pritchard *et al*, 2006; Bartlett *et al*, 2008, 2010; Munro *et al*, 2010). Ch17CEP duplication is the only predictive marker of anthracycline response providing consistent results across all trials (Bartlett *et al*, 2009). Duplication of Ch17CEP, however, does not immediately identify a functional link to a specific gene pathway. The chromosome enumeration probe (CEP) binds a non-coding pericentromeric *α*-satellite repeat on chromosome 17 that can both enumerate chromosome copy number and identify amplification of this region within the chromosome (without chromosome duplication) (Marchio *et al*, 2009).

In this study, we assessed CEPs for chromosomes 1, 7, 11 and 18 by fluorescent *in situ* hybridisation (FISH) to confirm whether Ch17CEP duplication is a surrogate marker of CIN in the context of anthracycline response. This, together with data previously collected for chromosome 17, was used to assess whether CIN is a potential predictive marker of anthracycline response in the BR9601 clinical trial.

## Patients and Methods

### Patients

The BR9601 study recruited 374 pre- and post-menopausal women with completely excised, histologically confirmed breast cancer who had a clear indication for adjuvant chemotherapy. Patients were randomised between the standard arm of eight cycles of CMF (i.v. cyclophosphamide 750 mg m^−2^, methotrexate 50 mg m^−2^ and 5-fluorouracil 600 mg m^−2^) given every 21 days and E-CMF (four cycles of epirubicin 100 mg m^−2^ every 21 days followed by four cycles of the same CMF regimen) (Poole *et al*, 2006); their characteristics are shown in Table 1. The protocol was approved by central and local ethics committees, and each patient provided written informed consent before randomisation. The primary outcomes of the BR9601 study were RFS and OS, and results were published in a joint analysis with the NEAT trial (Poole *et al*, 2006).

For the current analysis, following approval by central ethics committee, tissue blocks were retrieved from 322 cases (85.8%). Triplicate tissue microarrays (TMAs) were constructed following review by a pathologist (JST) according to current guidelines (Leyland-Jones *et al*, 2008).

### Fluorescent *in situ* hybridisation

Fluorescent *in situ* hybridisation was performed using centromeric probes for chromosomes 1, 7, 11 and 18 (Abbott Molecular, Maidenhead, UK), as previously described (Ellis *et al*, 2000; Bartlett *et al*, 2001). Chromosomes 1 and 11 were labelled with SpectrumOrange whereas chromosomes 7 and 18 were labelled with SpectrumGreen allowing dual colour FISH to be performed with chromosomes 1 and 7, and 11 and 18. Results for chromosome 17 were available from a previous analysis using the triple-colour probe for HER2/TOP2A/CHR17 (Bartlett *et al*, 2008). Centromeric duplication (CEP duplication) was defined as a chromosome CEP copy number >1.86, as previously defined for chromosome 17 (Watters *et al*, 2003; Bartlett *et al*, 2010).

### Defining chromosome instability

Signals for each chromosome were counted in intact non-overlapping nuclei. In total, 20–40 nuclei were counted in each tumour sample for chromosomes 1, 7, 11 and 18 (Watters *et al*, 2003) with 93–99% of samples having results for at least 20 nuclei for each chromosome. The percentage CIN for each tumour was defined by first calculating the percentage of nuclei with a CEP signal number different to the modal number for each individual chromosome (CIN % by chromosome=chromosome CIN%), and then calculating the mean CIN percentage of all chromosomes analysed (CIN % by tumour=tumour CIN%), as defined by Lengauer *et al* (1997). High tumour CIN% was defined as a mean tumour CIN percentage higher than the upper quartile; all other tumours were classed as having low tumour CIN%.

### Statistics

The SPSS (v14; SPSS Inc., Chicago, IL, USA) statistical package was used for statistical analysis. Kaplan–Meier estimates of survival were used for analysis of relapse-free and overall survival. The Cox’s proportional hazard model was used to obtain hazard ratios for relapse or death. When comparing outcomes between the treatment arms within the two groups of patients identified by bio-marker expression; *P*-values were not calculated for sub-groups to avoid multiple testing and bias, where one group was much smaller than the other. The Cox model was instead used to identify statistically significant interactions (*P*<0.05) between chromosomal alterations and outcome on the different treatments (treatment by marker effect), in models that also included chromosome status (marker effect) and treatment, as covariates.

## Results

### Correlation of CIN and clinicopathological parameters

Analysis of chromosomes 1, 7, 11, 17 and 18 was successful in 72.9%, 72.9%, 82.6%, 93.8% and 82.6% of cases, respectively. Fluorescent *in situ* hybridisation analysis of chromosome 17 was performed for an earlier study; therefore, the percentage of successfully analysed cases was lower for chromosomes 1, 7, 11 and 18 owing to a loss of cores on the TMAs. The percentage CIN for individual chromosomes (Table 2) was used to calculate the mean tumour CIN% for 86.3% (278) of tumour samples with the tumour CIN% ranging from 26.8% to 64.0% (median interquartile range: 40.0–47.3%). Tumours with over 47.3% CIN (above the upper quartile) were defined as having high tumour CIN% with all other tumours being classed as having low tumour CIN%. High tumour CIN% was correlated (Pearson’s χ^2^) with high pathological grade (*P*=0.008), HER2 amplification (*P*=2.89e-5), and Ch17CEP duplication (*P*=3.68e-7) but was not associated with size, nodal status, ER or proliferation (measured by Ki67; Supplementary Table S1).

### Tumour CIN% as a marker for RFS and OS

**Figure 1:**
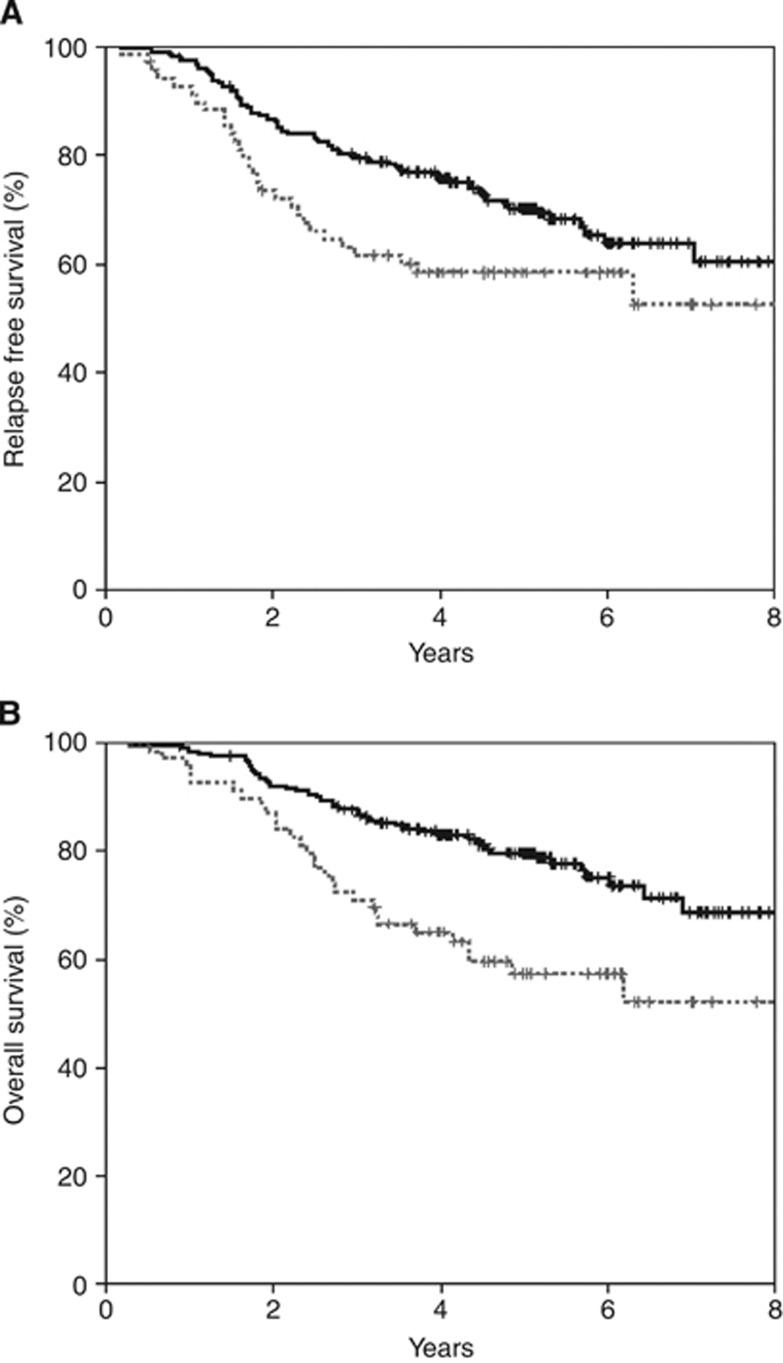
Relapse-free and overall survival for high tumour CIN- (dashed lines) *vs* low tumour CIN- (solid lines)treated cases. (**A**) Relapse-free survival. (**B**) Overall survival.

The prognostic significance of tumour CIN% in this study was first tested on the entire patient cohort, irrespective of allocated adjuvant chemotherapy. High tumour CIN% was associated with a markedly reduced RFS (Figure 1A; *P*=0.0011) and OS (Figure 1B; *P*=0.04). In univariate analysis, patients with a high tumour CIN% had a 1.58-fold (95% CI: 1.02–2.46) increased risk of dying from breast cancer and 2.13-fold (95% CI: 1.34–4.56) increased risk of relapse when compared with those with a low tumour CIN%. In multivariate analysis, high tumour CIN% was not an independent predictor for RFS (HR: 1.28, 95% CI: 0.74–2.209) but for OS there was a strong trend that did not reach statistical significance (HR: 1.76, 95% CI: 0.99–3.13, *P*=0.054).

### Tumour CIN% as a biological marker for anthracycline therapy

**Figure 2:**
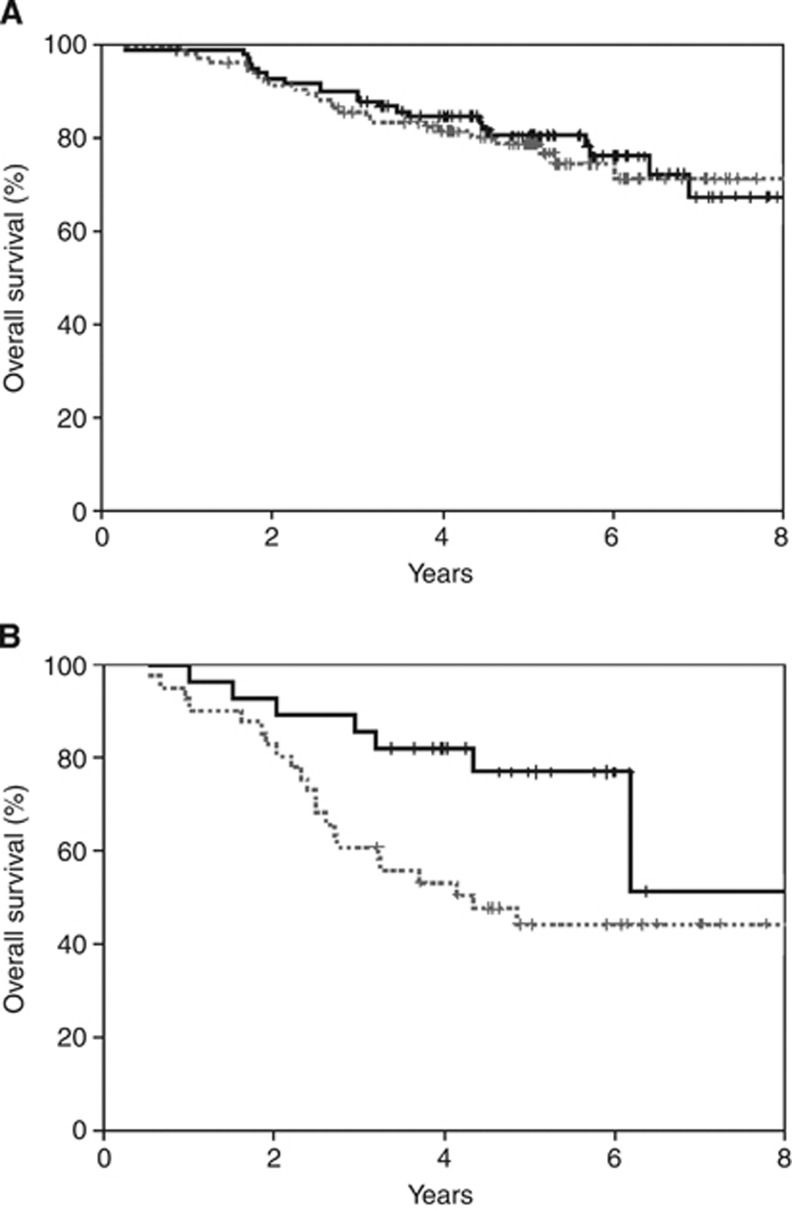
Overall survival for E-CMF- (solid lines) *vs* CMF- (dashed lines)treated cases. (**A**) Low tumour CIN% cases (see text). (**B**) High tumour CIN% cases (see text).

In univariate analysis, patients with high tumour CIN% had a decreased risk of death (HR: 0.39, 95% CI: 0.17–0.92) and a trend towards a decreased risk of relapse (HR: 0.46, 95% CI: 0.20–1.05) when treated with E-CMF compared with CMF alone (Figure 2A and B). No apparent benefit of E-CMF *vs* CMF was noted in patients with low tumour CIN% for either RFS or OS. In a multivariate analysis with adjustment for size, nodal status, ER, pathological grade, HER2, Ch17CEP duplication, Ki67, Ch17CEP*TREAT (treatment by marker interaction for Ch17CEP and treatment), tumour CIN%, TREAT (treatment) and tumour CIN%*TREAT showed only nodal status (for RFS and OS), and ER and high tumour CIN% (for OS but not RFS) to be statistically significant. There was a trend for tumour CIN%*TREAT for OS (HR: 0.31, 95% CI: 0.09–1.07, *P*=0.063), however, no significant association with RFS (HR: 0.55, 95% CI: 0.17–1.74, *P*=0.305) was apparent (Table 3).

### Individual chromosome analysis

Exploratory analyses were conducted using the copy number data for the individual chromosomes. Low Ch1CEP was associated with a reduced OS (HR: 1.66, 95% CI: 1.02–2.70, *P*=0.04) but not RFS (HR: 1.29, 95% CI: 0.82–2.01). No association with RFS and OS was noted for Ch7CEP, Ch11CEP, Ch17CEP or Ch18CEP (*P*>0.05).

Subsequent exploratory analyses assessed the effect of individual CEP duplication on RFS and OS between patients treated with E-CMF and those treated with CMF alone (Supplementary Table S2 and Supplementary Figure 1A and B). In a multivariate analysis and adjustment for size, nodal status, ER, pathological grade, HER2, Ch17CEP, Ki67, Ch17CEP*TREAT, Ch7CEP, TREAT and Ch7CEP*TREAT, the hazard ratio for the Ch7CEP treatment marker effect is 0.27 (95% CI: 0.08–0.96, *P*=0.042) for OS and 0.23 (95% CI: 0.07–0.75, *P*=0.015) for RFS (Supplementary Table S3).

## Discussion

In this study, we investigated the role of tumour CIN% in breast carcinomas recruited into the BR9601 trial. We have shown high tumour CIN% to be correlated to Ch17CEP duplication suggesting that Ch17CEP could be acting as a surrogate marker for CIN, therefore giving a more mechanistic/functional explanation to previous results (Bartlett *et al*, 2009). Furthermore, we have demonstrated high tumour CIN% to be a potential prognostic marker for OS and RFS in this cohort of breast cancer patients. This supports previous studies that have shown CIN to be associated with a poor prognosis in a number of solid tumours including breast cancer (Carter *et al*, 2006). Carter *et al* (2006) characterised CIN using a CIN70 gene signature containing genes known to be involved in processes such as chromosome segregation, cell cycle checkpoints and DNA replication. Assessment of CIN70 in the OV01 ovarian cancer clinical trial showed increased expression of CIN genes to be associated with both resistance to taxanes and sensitivity to carboplatin suggesting the predictive value of CIN may be drug dependent (Swanton *et al*, 2009). In this study, we have shown a trend towards significance for an increased benefit from the addition of anthracycline in patients with high CIN. The small number of patients in the high CIN group meant the test was statistically underpowered and therefore should be regarded as hypothesis generating and tested on a larger cohort of patients.

Exploratory analysis of the individual chromosomes highlighted Ch7CEP as another candidate marker of response to anthracyclines. In patients with low Ch7CEP, there is a 62.6% reduction in the risk of death when treated with anthracyclines compared with CMF. The association between low levels of Ch7CEP and response to anthracyclines is interesting; however, this study is underpowered and this would require testing in a large cohort.

Our findings demonstrate that Ch17CEP is associated with CIN. High tumour CIN% is of prognostic value and may be able to predict response to anthracycline-based therapy, although testing in a larger cohort would be required to confirm this.

## Figures and Tables

**Table 1:** Patient characteristics from the BR9601 trial

	**BR9601**	**TMA**
Number	374	322
Age (years)	50.6 (22.7–76.0)	50.6 (26.2–76.0)
		
E-CMF	183 (48.9%)	158 (49.1%)
CMF	191 (51.1%)	164 (50.9%)
		
*Size*		
<2.0 cm	123 (32.9%)	105 (32.7%)
2.0–5.0 cm	226 (60.4%)	197 (61.1%)
>5.0 cm	25 (6.7%)	20 (6.2%)
		
*Nodes*		
0	48 (12.8%)	44 (13.6%)
1–3	214 (57.3%)	180 (56.0%)
⩾4	112 (29.9%)	98 (30.4%)
		
*Grade*		
1	22 (5.9%)	22 (6.9%)
2	126 (33.7%)	109 (30.4%)
3	210 (56.1%)	189 (58.6%)
Unknown	16 (4.2%)	2 (0.6%)
		
NPI	5.30 (4.50–5.61)	5.30 (4.50–5.60)
		
*ER status*		
Positive	202 (62.9%)	176 (62.6%)
Negative	119 (37.1%)	105 (37.4%)
Unknown	53	40

Abbreviations: CMF=cyclophosphamide, methotrexate and 5-fluorouracil; E-CMF=epirubicin CMF; ER=oestrogen receptor; NPI=Nottingham Prognostic Index; TMA=tissue microarray.

**Table 2:** Average and median CIN for individual chromosomes

**Chromosome**	**Average chromosomal CIN (%)**	**Median CIN (interquartile range) (%)**
CIN1	43.6	45.0 (36.0–50.0)
CIN7	43.6	45.0 (37.5–50.0)
CIN11	42.3	43.8 (37.5–47.5)
CIN17	45.8	46.7 (40.0–52.1)
CIN18	42.0	44.4 (35.7–48.4)

Abbreviation: CIN=chromosome instability.

**Table 3:** Multivariate analysis of tumour CIN% and treatment interaction

	**OS**	***P*** **-value**	**RFS**	***P*** **-value**
TREAT	0.653 (0.294–1.448)	0.294	0.644 (0.322–1.285)	0.212
Size (>20 mm)	0.849 (0.465–1.549)	0.593	1.074 (0.618–1.869)	0.799
Nodal status (positive *vs* negative)	5.890 (1.756–19.757)	0.004	6.954 (2.082–23.225)	0.002
ER	0.461 (0.246–0.864)	0.016	0.653 (0.372–1.147)	0.138
Pathological grade (grade1/2 *vs* 3)	1.956 (0.920–4.160)	0.081	1.478 (0.786–2.780)	0.226
HER2 amplification	1.510 (0.838–2.722)	0.170	1.334 (0.773–2.301)	0.301
Ch17CEP	0.811 (0.355–1.855)	0.620	1.249 (0.609–2.562)	0.543
High Ki67 (>13%)	0.860 (0.458–1.615)	0.638	1.053 (0.597–1.856)	0.859
High tumour CIN%	2.864 (1.334–6.151)	0.007	1.615 (0.792–3.292)	0.188
Ch17CEP*TREAT	1.618 (0.514–5.093)	0.410	1.021 (0.361–2.883)	0.969
Tumour CIN%*TREAT	0.308 (0.089–1.067)	0.063	0.545 (0.171–1.736)	0.305

Abbreviation: CIN%=percentage chromosome instability; ER=oestrogen receptor; OS=overall survival; RFS=relapse-free survival; TREAT=treatment.

## References

[bib1] Bartlett JM, Desmedt C, Munro A, O’Malley FP, Larsimont D, Di Leo A, Cameron DA, Isola J, Shepherd L, Twelves CJ, Pritchard KI. for the HER (2009) Chromosome 17 polysomy: a unifying hypothesis underlying benefit from adjuvant anthracyclines? Cancer Res 69: 6059

[bib2] Bartlett JMS, Going JJ, Mallon E, Watters AD, Reeves JR, Stanton PD, Richmond J, Donald B, Ferrier R, Cooke TG (2001) Evaluating HER2 amplification and overexpression in breast cancer. J Pathol 195: 422–428 11745673 10.1002/path.971

[bib3] Bartlett JMS, Munro AF, Cameron DA, Thomas JS, Prescott RJ, Twelves C (2008) Type I receptor tyrosine kinase profiles identify patients with enhanced benefit from anthracyclines in the BR9601 adjuvant breast cancer chemotherapy trial. J Clin Oncol 26: 1–9 10.1200/JCO.2007.14.6597 18768436

[bib4] Bartlett JM, Munro AF, Dunn JA, McConkey C, Jordan S, Twelves CJ, Cameron DA, Thomas J, Campbell FM, Rea DW, Provenzano E, Caldas C, Pharoah P, Hiller L, Earl H, Poole CJ (2010) Predictive markers of anthracycline benefit: a prospectively planned analysis of the UK National Epirubicin Adjuvant Trial (NEAT/BR9601). Lancet Oncol 11: 266–274 20079691 10.1016/S1470-2045(10)70006-1

[bib5] Carter SL, Eklund AC, Kohane IS, Harris LN, Szallasi Z (2006) A signature of chromosomal instability inferred from gene expression profiles predicts clinical outcome in multiple human cancers. Nat Genet 38: 1043–1048 16921376 10.1038/ng1861

[bib6] Ellis IO, Dowsett M, Bartlett J, Walker R, Cooke T, Gullick W, Gusterson B, Mallon E, Barrett Lee P (2000) Recommendations for HER2 testing in the UK. J Clin Pathol 53: 890–892 11265171 10.1136/jcp.53.12.890 PMC1731136

[bib7] Lengauer C, Kinzler KW, Vogelstein B (1997) Genetic instability in colorectal cancers. Nature 386: 623–627 9121588 10.1038/386623a0

[bib8] Leyland-Jones BR, Ambrosone CB, Bartlett J, Ellis MJC, Enos RA, Raji A, Pins MR, Zujewski JA, Hewitt SM, Forbes JF, Abramovitz M, Braga S, Cardoso F, Harbeck N, Denkert C, Jewell SD (2008) Recommendations for collection and handling of specimens from group breast cancer clinical trials. J Clin Oncol 26: 5638–5644 18955459 10.1200/JCO.2007.15.1712 PMC2651095

[bib9] Marchio C, Lambros MB, Gugliotta P, Di Cantogno LV, Botta C, Pasini B, Tan DS, Mackay A, Fenwick K, Tamber N, Bussolati G, Ashworth A, Reis-Filho JS, Sapino A (2009) Does chromosome 17 centromere copy number predict polysomy in breast cancer? A fluorescence *in situ* hybridization and microarray-based CGH analysis. J Pathol 219: 16–24 19670217 10.1002/path.2574

[bib10] Miyoshi Y, Iwao K, Ikeda N, Egawa C, Noguchi S (2002) Acceleration of chromosomal instability of BRCA1-associated hereditary breast cancers by p53 abnormality. Breast J 8: 77–80 11896751 10.1046/j.1524-4741.2002.08201.x

[bib11] Munro AF, Cameron DA, Bartlett JMS (2010) Targeting anthracyclines in early breast cancer: new candidate predictive biomarkers emerge. Oncogene 29: 5231–5240 20676126 10.1038/onc.2010.286

[bib12] Poole CJ, Earl HM, Hiller L, Dunn JA, Bathers S, Grieve RJ, Spooner DA, Agrawal RK, Fernando IN, Brunt AM, O’Reilly SM, Crawford SM, Rea DW, Simmonds P, Mansi JL, Stanley A, Harvey P, McAdam K, Foster L, Leonard RCF, Twelves CJ, The NEAT Investigators and the SCTBG (2006) Epirubicin and cyclophosphamide, methotrexate, and fluorouracil as adjuvant therapy for early breast cancer. N Engl J Med 355: 1851–1862 17079759 10.1056/NEJMoa052084

[bib13] Pritchard KI, Shepherd LE, O’Malley FP, Andrulis IL, Tu D, Bramwell VH, Levine MN, The National Cancer Institute of Canada Clinical Trials Group (2006) HER2 and responsiveness of breast cancer to adjuvant chemotherapy. N Engl J Med 354: 2103–2111 16707747 10.1056/NEJMoa054504

[bib14] Smid M, Hoes M, Sieuwerts A, Sleijfer S, Zhang Y, Wang Y, Foekens J, Martens J (2010) Patterns and incidence of chromosomal instability and their prognostic relevance in breast cancer subtypes. Breast Cancer Res Treat 128: 23–30 20632083 10.1007/s10549-010-1026-5

[bib15] Swanton C, Nicke B, Schuett M, Eklund AC, Ng C, Li Q, Hardcastle T, Lee A, Roy R, East P, Kschischo M, Endesfelder D, Wylie P, Kim SN, Chen JG, Howell M, Ried T, Habermann JK, Auer G, Brenton JD, Szallasi Z, Downward J (2009) Chromosomal instability determines taxane response. PNAS 106: 8671–8676 19458043 10.1073/pnas.0811835106 PMC2688979

[bib16] Takami S, Kawasome C, Kinoshita M, Koyama H, Noguchi S (2001) Chromosomal instability detected by fluorescence *in situ* hybridization in Japanese breast cancer patients. Clin Chim Acta 308: 127–131 11412824 10.1016/s0009-8981(01)00473-9

[bib17] Walther A, Houlston R, Tomlinson I (2008) Association between chromosomal instability and prognosis in colorectal cancer: a meta-analysis. Gut 57: 941–950 18364437 10.1136/gut.2007.135004

[bib18] Watters AD, Going JJ, Cooke TG, Bartlett JM (2003) Chromosome 17 aneusomy is associated with poor prognostic factors in invasive breast carcinoma. Breast Cancer Res Treat 77: 109–114 12602909 10.1023/a:1021399923825

