# Supplementary material for: Chromosome instability and benefit from adjuvant anthracyclines in breast cancer
Source: Br J Cancer. 2012 May 29;107(1):71–4. doi: 10.1038/bjc.2012.232 (PMC3389422; doi:10.1038/bjc.2012.232)
Supplement: Supplementary Information [file bjc2012232x1.doc]

Supplementary Data:

Figure S1: Relapse Free Survival by Ch7CEP and Treatment

A) Low Ch7CEP


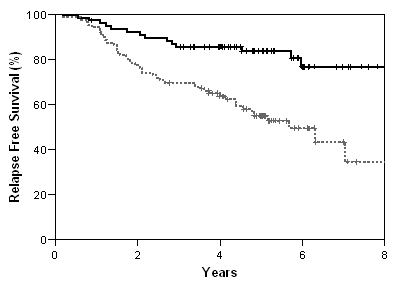


B) High Ch7CEP


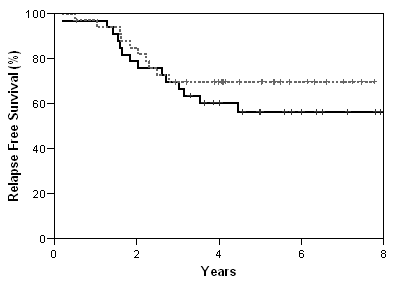


Figure S1: Relapse free survival for E-CMF (solid lines) versus CMF (dashed lines) treated cases. Panel A: Low Ch7CEP cases (see text). Panel B: High Ch7CEP cases (see text).

Table S1: Crosstabulation of CIN with clinicopathological markers

|  | **Low tumour CIN%** | **High tumour CIN%** | **p-value** |
| --- | --- | --- | --- |
| **Size:**  <20mm  >20mm | 63 (78.8%)  140 (74.1%) | 17 (21.3%)  49 (25.9%) | 0.415 |
| **Nodes:**  Negative  1-3 nodes  >4 nodes | 33 (82.5%)  115 (75.2%)  56 (70.9%) | 7 (17.5%)  38 (24.8%)  23 (29.1%) | 0.384 |
| **ER status:**  Positive  Negative | 112 (77.8%)  69 (71.9%) | 32 (22.2%)  27 (28.1%) | 0.298 |
| **Pathological Grade:**  Grade 1  Grade 2  Grade 3 | 16 (100.0%)  72 (81.8%)  120 (70.2%) | 0 (0%)  16 (18.2%)  51 (29.8%) | 0.008 |
| **Proliferation:**  Low Ki67  High Ki67 | 122 (77.7%)  82 (71.3%) | 35 (22.3%)  33 (28.7%) | 0.228 |
| **HER2:**  Non-amplified  Amplified | 167 (81.1%)  34 (84.8%) | 39 (18.9%)  28 (45.2%) | 2.89e-5 |
| **Ch17CEP:**  Low  High | 142 (85.5%)  59 (57.8%) | 24 (14.5%)  43 (42.2%) | 3.68e-7 |

Table S2: Hazard ratios for OS and RFS for exploratory analysis of individual chromosomes.

|  | | OS | p-value | RFS | p-value |
| --- | --- | --- | --- | --- | --- |
| Ch1CEP | Low | 0.460  (0.220-0.963) | 0.398 | 0.461  (0.246-0.863 | 0.578 |
|  | High | 0.700  (0.344-1.422) |  | 0.597  (0.299-1.193) |  |
| Ch7CEP | Low | 0.374  (0.192-0.727) | 0.023 | 0.367  (0.189-0.714) | 0.004 |
|  | High | 1.323  (0.557-3.143) |  | 1.377  (0.580-3.268) |  |
| Ch11CEP | Low | 0.717  (0.420-1.226) | 0.493 | 0.622  (0.379-1.019) | 0.806 |
|  | High | 0.508  (0.179-1.444) |  | 0.577  (0.235-1.147) |  |
| Ch17CEP | Low | 0.751  (0.409-1.378) | 0.329 | 0.703  (0.409-1.206) | 0.335 |
|  | High | 0.490  (0.251-0.959) |  | 0.480  (0.260-0.885) |  |
| Ch18CEP | Low | 0.689  (0.411-1.155) | 0.556 | 0.603  (0.377-0.964) | 0.848 |
|  | High | 0.487  (0.134-1.772) |  | 0.574  (0.183-1.805) |  |

Table S3: Multivariate analysis of Ch7CEP and treatment interaction

|  | OS | p-value | RFS | p-value |
| --- | --- | --- | --- | --- |
| Treatment (TREAT) | 0.340  (0.139-0.829) | 0.018 | 0.345  (0.153-0.777) | 0.010 |
| Size (>20mm) | 0.849  (0.457-1.580) | 0.606 | 1.004  (0.569-1.773) | 0.989 |
| Nodal Status  (positive vs negative) | 5.191  (1.511-17.831) | 0.009 | 6.578  (1.941-22.291) | 0.002 |
| ER | 0.620  (0.316-1.217) | 0.165 | 0.792  (0.429-1.461) | 0.455 |
| Pathological Grade (Grade1/2 vs 3) | 1.996  (0.934-4.262) | 0.074 | 1.852  (0.953-3.602) | 0.069 |
| HER2 amplification | 1.611  (0.861-3.014) | 0.136 | 1.261  (0.702-2.265) | 0.438 |
| Ch17CEP | 1.055  (0.489-2.274) | 0.891 | 1.345  (0.678-2.672) | 0.396 |
| High Ki67  (>13%) | 1.040  (0.538-2.009) | 0.908 | 1.099  (0.605-1.997) | 0.757 |
| Ch7CEP | 0.535  (0.217-1.316) | 0.173 | 0.492  (0.203-1.191) | 0.116 |
| Ch17CEP*TREAT | 1.045  (0.339-3.216) | 0.939 | 0.803  (0.290-2.226) | 0.673 |
| Tumour  Ch7CEP*TREAT | 3.647  (1.049-12.674) | 0.042 | 4.399  (1.335-14.496) | 0.015 |
